# The mechanism and application of *Scrophulariae Radix* in the treatment of endocrine disorders: focusing on thyroid diseases and diabetes mellitus

**DOI:** 10.3389/fendo.2025.1684744

**Published:** 2025-11-21

**Authors:** Jiaying Song, Jiuwei Li, Huishan Shi, Huayuan Liu, Yuehan Qu, Furong Wang

**Affiliations:** 1 College of Traditional Chinese Medicine, Shandong University of Traditional Chinese Medicine, Jinan, Shandong, China; 2 Medical College, Shandong University of Traditional Chinese Medicine, Jinan, Shandong, China

**Keywords:** *Scrophulariae Radix*, endocrine disorders, thyroid diseases, diabetes mellitus, pharmacological effects, mechanisms

## Abstract

Endocrine disorders are prevalent worldwide, with particularly high incidence rates of thyroid diseases and diabetes mellitus (DM), which impose a substantial burden on healthcare systems. Life-long medication is often required to manage these conditions. *Scrophulariae Radix* is a traditional Chinese medicine. It contains various chemical components, such as iridoids, phenylpropanoids, and organic acids. These components show anti-inflammatory, antioxidative, and immunomodulatory effects. They have potential to treat endocrine disorders. They are especially effective for thyroid diseases and DM. This review aims to summarize the mechanisms and effects through which *Scrophulariae Radix* affects thyroid diseases and DM, providing further guidance for clinical treatment.

## 1 Introduction

Endocrine disorders refer to a group of conditions that disrupt the normal functioning of the endocrine system, including thyroid, pancreatic, and adrenal diseases.

Thyroid diseases are particularly common and include hyperthyroidism, hypothyroidism, goiter, thyroiditis, thyroid nodules, and thyroid cancer. These conditions present with diverse symptoms, such as weight loss, accelerated heart rate, anxiety, and exophthalmos (1). With changes in modern lifestyles, the incidence of thyroid diseases has been increasing annually. The prevalence rate in the general population is 10–15%, but this figure is significantly higher in elderly individuals, reaching up to 25% in some groups (2). Clinically, thyroid diseases are treated with medications, radioactive iodine therapy, and surgery; however, these approaches are often associated with side effects and complications, making long-term and effective disease control challenging (3, 4). *Scrophulariae Radix* is traditionally believed to possess detoxifying and lump-resolving properties, suggesting potential therapeutic benefits for treating thyroid diseases.

Diabetes mellitus (DM) is one of the most common metabolic diseases and is caused by defective insulin secretion or impaired insulin action. It includes type 1 diabetes, type 2 diabetes, and gestational diabetes, of which type 2 diabetes is the most common (5, 6). Symptoms include polyuria, polydipsia, polyphagia, and weight loss (7). The etiology of diabetes is related to genetic factors or environmental factors, such as obesity, urbanization, gene mutations, and a lack of physical exercise, and its incidence and mortality rates are increasing annually. It is expected to become the seventh leading cause of death worldwide by 2030 (7, 8). Owing to long-term unstable blood sugar levels, complications such as cardiovascular disease, neuropathy, eye disease, and kidney disease may occur (9). The mortality rate of diabetes complications is as high as 23.2% (10). Currently, modern medical treatments for diabetes include insulin, insulin sensitizers, and α-glucosidase inhibitors, but the disease is prone to relapse.

*Scrophularia Radix* is the dried root of *Scrophularia ningpoensis* Hemsl., a plant of the Scrophulariaceae family. *Scrophularia Radix* was first recorded in Shennong’s Herbal Classic and is widely used in traditional Chinese medicine (TCM) (11). *Scrophularia Radix* is well known for its heat-clearing, yin-nourishing and detoxifying effects. For thousands of years, *Scrophularia Radix* has been used to treat a variety of diseases, including sore throat, abscesses, carbuncles and constipation (12). *Scrophularia Radix* tastes bitter, sweet and salty and is cold in nature. It can be used clinically to treat hypertension, diabetes, cancer and inflammatory diseases (11, 13). From the perspective of traditional Chinese medicine, *Scrophularia Radix* is extremely common in nature and removes fever, eliminates evil, nourishes yin and cools the body. It may be effective in treating thyroid diseases and diabetes. *Scrophularia Radix* has potential in the treatment of thyroid diseases and diabetes through a multicomponent and multitarget mechanism of action.

This article reviews the chemical components, pharmacological effects, and mechanisms of action of *Scrophularia ningpoensis* in the treatment of thyroid diseases and diabetes. It provides a reference for the clinical application of *Scrophularia ningpoensis* in endocrine diseases. It focuses especially on thyroid diseases and diabetes. It also supports the further development of *Scrophularia ningpoensis*.

## 2 Major chemical components and pharmacological effects of *Scrophulariae Radix*

### 2.1 Major chemical components

*Scrophulariae Radix*, a well-known TCM, contains various active constituents, including iridoids, phenylpropanoids, organic acids, volatile oils, steroids, carbohydrates, flavonoids, alkaloids, and phenols. These compounds exhibit significant pharmacological effects in the treatment of thyroid diseases and DM (14). The representative components of *Scrophulariae Radix* and their mechanisms of action are summarized in **Table 1**. The following section provides a detailed review of the major chemical constituents and their pharmacological activities in *Scrophulariae Radix*.

**Table 1:** Representative *Scrophulariae Radix* and their mechanisms of action.

*Scrophulariae Radix* subclass	Name of *Scrophulariae Radix*	Structure of *Scrophularia Radixe*	Function of *Scrophularia Radixe*	Mechanism of action	References
Iridoid Glycosides	Harpagide	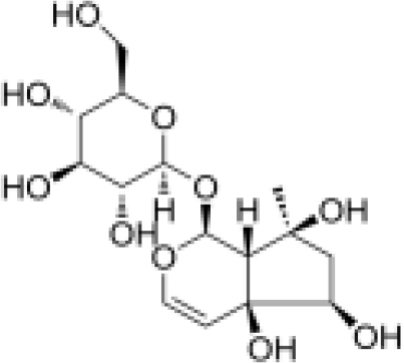	Anti-inflammation Neuroprotection Immunomodulation	Promoted cell migration reduced TNFα secretion induced mRNA expression of certain proteins associated with leukocyte migration in undifferentiated THP-1 cells	(52)
Iridoid Glycosides	Harpagoside	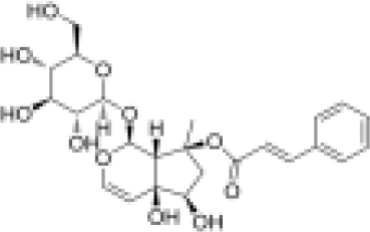	Anti-inflammation Immune regulation Impact on metabolism	Inhibited the nuclear translocation of NF-κB pathway and degrade its inhibitory protein IκBAα, inhibited the expression of downstream pro-inflammatory factors (IL-6), and realize its anti-inflammatory effect. Reduce lipid peroxidation in brain tissue, increase glutathione and superoxide dismutase activity, and reduce oxidative damage. Activation of PPAR-γ inhibits chronic inflammation in adipose tissue, which may have potential efficacy on metabolism.	(53, 54)
Iridoid Glycosides	catalpol	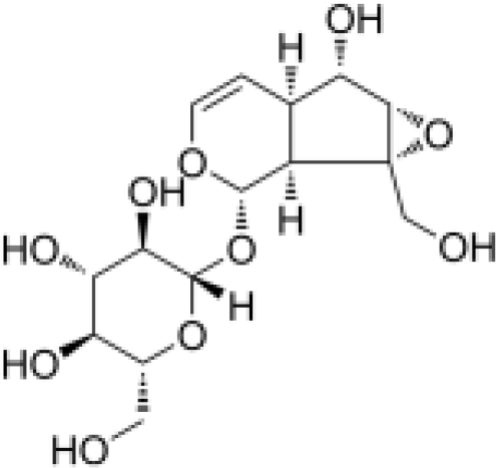	Anti-inflammation Antioxidant Hypoglycemic effect Anti-tumor Kidney protection	Inhibited NF-κB, TLR4/MyD88 and p38 MAPK signaling pathway Reduced the release of pro-inflammatory factors (TNF-α, IL-6, IL-1β) Activated AMPK/mTOR pathway Inhibited AGEs/RAGE signaling to reduce oxidative stress and inflammation in diabetic nephropathy Inhibited NOX4 expression to reduce the level of oxidative stress Inhibited TGF-β1 pathway, reduce renal fibrosis and inflammation	(26, 55–58)
Iridoid Glycosides	Loganic acid	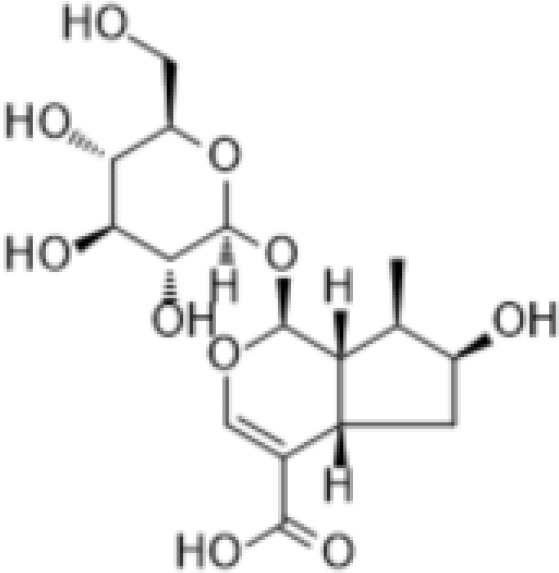	Hypoglycemic effect Antioxidation Anti-inflammation	Inhibition of acetylcholinesterase (AChE) and α-glucosidase Activated peroxisome proliferator-activated receptor α (PPARα) pathway, regulate lipid metabolism and inflammatory response	(59, 60)
Iridoid Glycosides	Geniposide	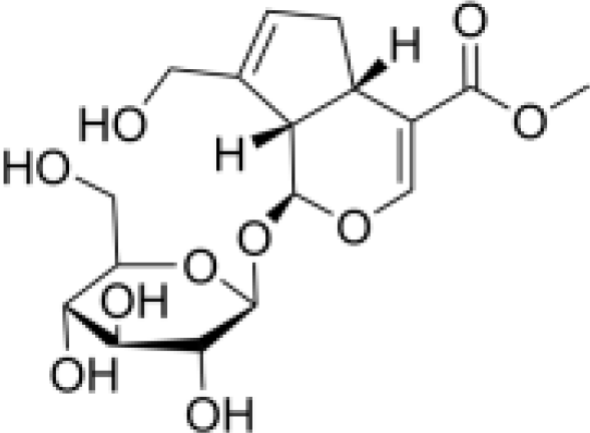	Anti-inflammation Hypoglycemic effect Metabolic regulation	Inhibited EGFR/PI3K/AKT signaling pathway, reduced the released of inflammatory factors IL-1β, IL-6, TNF-α Improved lipid and glucose metabolism, promoted adipogenesis, improved insulin sensitivity by regulating PI3K-Akt signaling pathway, and fight against diabetes and its complications.	(61, 62)
Iridoid Glycosides	Aucubin	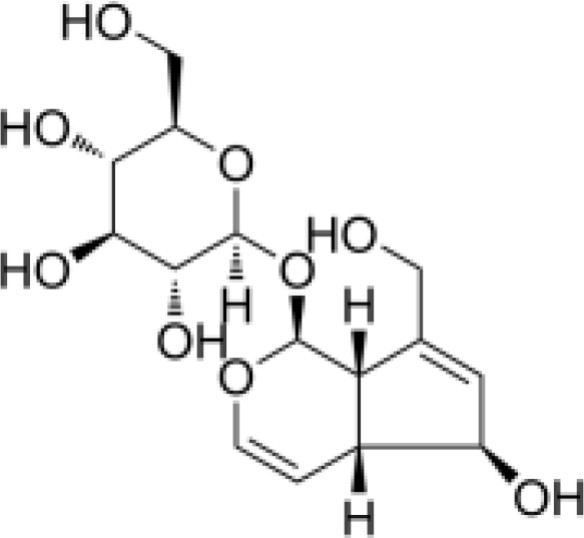	Antioxidation Anti-apoptosis Anti-inflammation	Scavenging free radicals, upregulating glutathione levels, inhibiting caspase cascade reactions (e.g. c-caspase-3/9) and PARP cleavage, and reducing oxidative stress-induced apoptosis. Inhibited IκBα degradation and p65 nuclear translocation, reduced pro-inflammatory factor release.	(63, 64)
phenylpropanoids	Angoroside C	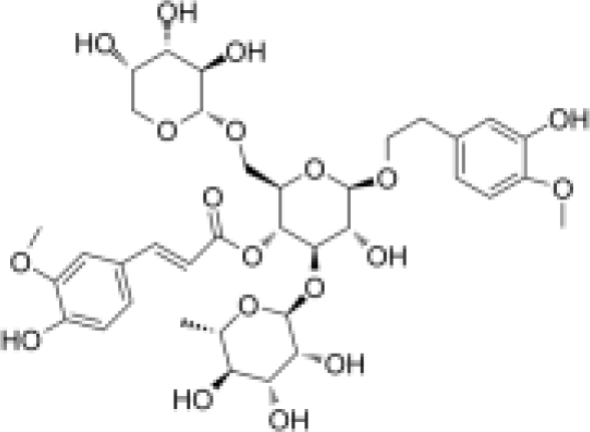	Anti-inflammation Anti-tumor	Inhibited COX-1 and COX-2 pathway, reduce the production of thromboxane B2 and prostaglandin E2. Inhibited the mRNA and protein expression of TNF-α in RAW264.7 cells to realize its anti-inflammatory and anti-tumor effects.	(65, 66)
phenylpropanoids	Acteoside	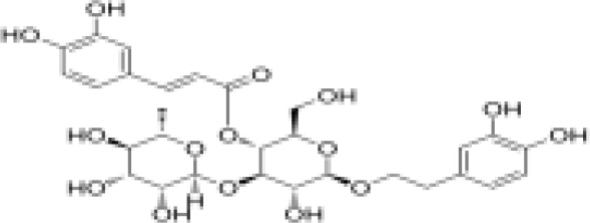	Anti-inflammation Antioxidation Anti-tumor Protect the retina	Activated NRf2, ARE signaling pathway to activate antioxidant enzymes Inhibit hepatocellular carcinoma cell proliferation, tube formation and cell migration Regulated PI3K/AKT signaling pathway to treat diabetic nephropathy and alleviate coke death.	(67–70)
phenylpropanoids	Caffeic acid	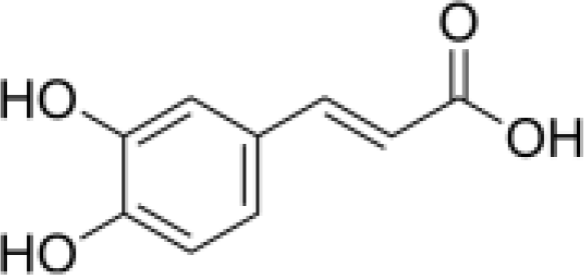	Anti-inflammation Metabolic regulation Hypoglycemic effect Antioxidant	Inhibited cyclooxygenase-2 (COX-2) and its product prostaglandin E (PGE), reduce the synthesis of IL-8, reduce intestinal inflammation Enhanced glucose transportation or inhibit gluconeogenesis. Inhibited the formation of ACE, alleviate diabetes complications.	(71, 72)
phenylpropanoids	Ferulic acid	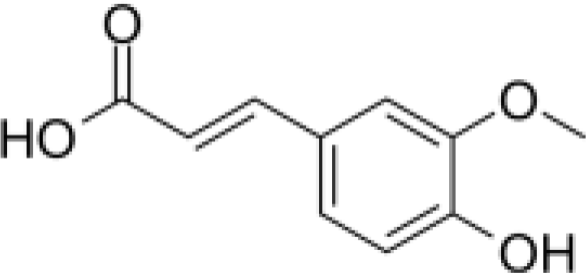	Antioxidation Anti-inflammation Hypoglycemic effect Anti-tumor Metabolic regulation	Scavenging free radicals, inhibiting the activity of lipid peroxidase SOD and catalase CAT to exert antioxidant effects. Inhibited LPS-TLR4-NF-κB and NF-κB-INOS-NO signaling pathway, thus reduced inflammation.	(73, 74)
phenylpropanoids	p-coumaric acid	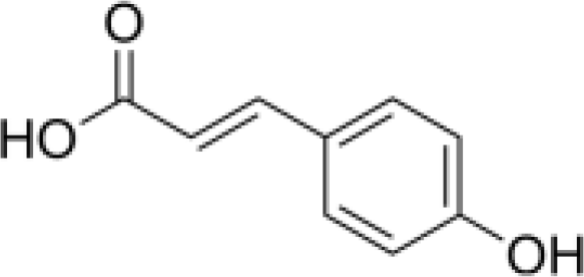	Antioxidation Anti-inflammation Hypoglycemic effect Metabolic regulation	Activated Nrf-2/HO-1 signaling pathway and enhanced the expression of intracellular antioxidant enzymes, thus scavenged free radicals and reduced oxidative stress. Inhibited the expression of pro-inflammatory factors (TNF-α, IL-6) and reduced the activation of NF-Κbd. Inhibited α-amylase activity to lower blood glucose and improved high-fat diet-induced insulin resistance and lipid metabolism disorders.	(75–78)
phenylpropanoids	Ursolic acid	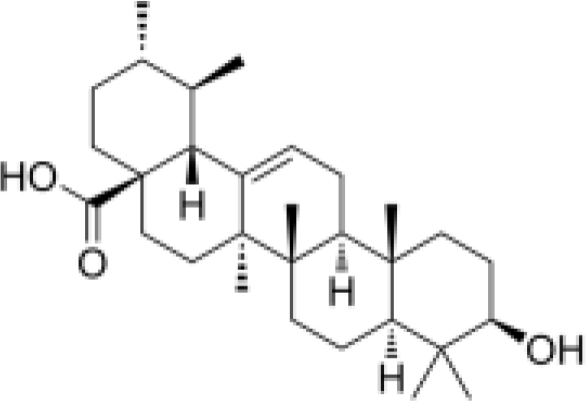	Anti-inflammation Immunity regulation Metabolic regulation Hypoglycemic effect	Inhibited mast cell TSLP and NF-κB pathway, reduced pro-inflammatory factors (TNF-α, IL-6) release. Upregulated superoxide dismutase and scavenge free radicals (SOD). Promoted mitochondrial biosynthesis and improved energy metabolism.	(79–81)
steroids	β-sitosterol	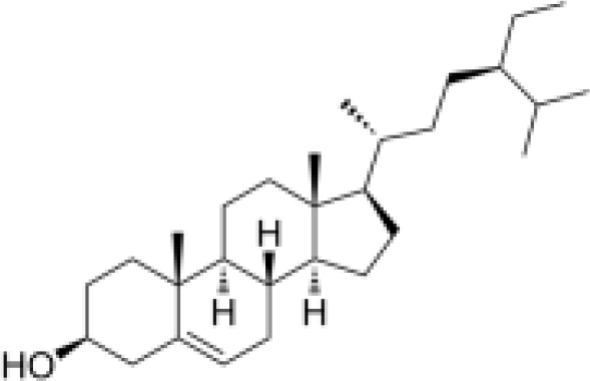	Anti-inflammation Metabolic regulation	Inhibited IKKβ/IκBα phosphorylation, block NF-κB nuclear translocation, reduced the production of pro-inflammatory factors (TNF-α, IL-6) Regulated the expression of PPARγ, *Scrophulariae Radix*EBP-1c, reduced fat accumulation and improved insulin sensitivity.	(82, 83)

#### 2.1.1 Iridoids

Iridoids are the most abundant and distinctive active chemical constituents in *Scrophulariae Radix*. They primarily consist of five-membered and six-membered rings and exhibit notable anticancer, anti-inflammatory, and hepatoprotective effects (15). Among these compounds, aucubin, geniposide, and catalpol are representative iridoids that are present mainly in glycosidic forms and release active compounds upon hydrolysis (16–24).

An et al. isolate a riboside from the stem bark of bovine polysaccharide. This compound inhibits the production of tumor necrosis factor-α (TNF-α), interleukin-1β (IL-1β), and interleukin-6 (IL-6). It also blocks the activation of NF-κB in lipopolysaccharide (LPS)-stimulated macrophages. As a result, it reduces tissue inflammation (25). Bhattamisra et al. report that riboside catalpol can activate antioxidant enzymes (such as superoxide dismutase (SOD) and catalase (CAT), reduce the level of oxidative stress in the body, and protect cells (26). Clinical studies have shown that Catalpol can inhibit the activity of thyroid peroxidase and treat thyroid diseases. Therefore, cyclic ene ether has important clinical value because of its anti-inflammatory, immunoregulatory and antioxidative effects (26).

#### 2.1.2 Phenylpropanoids

Phenylpropanoids are composed of a benzene ring and a propyl chain and include mainly coniferin and phenylethanol glycosides, which are abundant in *Scrophulariae Radix* and possess diverse biological activities, such as antioxidation, antitumor, and immunomodulation (27).

Coniferin helps scavenge free radicals, while phenylpropanoids increase the activity of antioxidant enzymes, protecting cells from oxidative damage (28, 29). In thyroid disease treatment, they may reduce inflammation and tissue damage by blocking pathways such as the NF-κB pathway (30). Phenylethanol glycosides also show promise for thyroid-related immunotherapy.

#### 2.1.3 Organic acids

Hippuric and caffeic acids are key organic acids in *Scrophulariae Radix* (31). They inhibit cyclooxygenase (COX) and lipoxygenase (LOX)) enzymes, lowering inflammatory mediator production (32). Caffeic acid also increases the activity of antioxidant enzymes, reducing the risk of free radicals and oxidative damage. Rosmarinic acid has been shown to improve thyroid structure and reduce inflammation in autoimmune thyroiditis models (33). These findings suggest that organic acids may offer therapeutic benefits for severe thyroid inflammation.

#### 2.1.4 Volatile oils

The volatile oils of *Scrophulariae Radix*, such as caryophyllene and eugenol, have strong antibacterial and analgesic effects (34, 35). Their lipid solubility allows them to disrupt microbial membranes, inhibiting pathogen growth (36). Caryophyllene binds to specific receptors in the nervous system, producing sedative and analgesic effects (37), which reduce thyroid-related symptoms in patients. Moreover, volatile oils have central inhibitory effects, alleviating anxiety and insomnia symptoms caused by thyroid and DM abnormalities.

#### 2.1.5 Steroids

Three steroidal components, namely, β-sitosterol, ursodeoxycholic acid, and carotene, have been isolated from *Scrophularia ningpoensis*. β-Sitosterol is known for its cholesterol-lowering, anti-inflammatory, antioxidant, immunomodulatory, antitumor, and wound healing effects (38). Ursodeoxycholic acid has choleretic, hepatoprotective, hypoglycemic, hypolipidemic, immunomodulatory, and antitumor effects (39, 40). Carotene has been shown to inhibit cancer cell proliferation, have antioxidant properties, protect blood vessels, and promote the proliferation of osteoblast-like cells (41–44).

#### 2.1.6 Carbohydrates

Carbohydrate components, such as *Scrophularia Radix*ceae polysaccharides, are composed of six monosaccharides, including galacturonic acid, glucose, mannose, rhamnose, galactose, and arabinose, and have antioxidant, anti-inflammatory, and antitumor effects (42).

*Scrophularia Radix*ceae *Radix* polysaccharides enhance immunity by promoting lymphocyte proliferation and enhancing macrophage phagocytosis. In addition, they can also assist in the prevention and treatment of thyroid immune diseases by activating natural killer (NK) cells (43, 44). *Scrophularia Radix*ceae polysaccharides also have antifatigue effects and can improve the symptoms of fatigue and weakness caused by metabolic disorders in patients with thyroid diseases and diabetes (45).

#### 2.1.7 Flavonoids

Flavonoids are widely present in nature and have various biological activities, such as liver protection, antitumor, and antioxidant effects (45, 46). Luteolin inhibits the proliferation and metastasis of cancer cells by inhibiting certain tumor growth factors and signaling pathways (such as the MAPK and PI3K/Akt pathways) (47, 48), providing a theoretical basis for the prevention and treatment of endocrine diseases.

#### 2.1.8 Alkaloids

Alkaloids in *Scrophulariae Radix*, such as genipin, act mainly on the nervous system and have sedative, antidepressant and antianxiety effects (49, 50). Genipin improves mood swings caused by thyroid dysfunction by regulating the release of neurotransmitters such as dopamine and serotonin (50). In addition, genipin also exerts a neuroprotective effect by inhibiting oxidative stress and reducing inflammatory responses (49). This helps to improve central nervous system symptoms caused by endocrine diseases.

#### 2.1.9 Phenols

Phenolic compounds such as chlorogenic acid have antioxidant, anti-inflammatory, and antibacterial effects (51). Chlorogenic acid inhibits thyroid peroxidase, helping regulate excess thyroid hormone production and managing hyperthyroidism (33). Its antioxidant action also protects cells by neutralizing free radicals (51).

## 3 Pharmacological effects

Studies on *Scrophulariae Radix* have highlighted its anti-inflammatory, antioxidant, immunomodulatory, cardiovascular protective, and antitumor effects (11).

### 3.1 Anti-inflammatory effects

Key components of *Scrophulariae Radix*, including iridoid glycosides such as aucubin and geniposide, show strong anti-inflammatory activity (84). Its extracts may regulate cytokines such as IL-6, IL-5, IL-13, IL-17, and IL-1 by influencing the NF-κB pathway (28). Phenylpropanoid glycosides also inhibit COX and LOX, reducing the levels of proinflammatory mediators such as prostaglandins. This finding indicates the potential for treating chronic inflammation (85). This potent anti-inflammatory activity is of particular relevance to the treatment of autoimmune thyroiditis (e.g., Hashimoto’s thyroiditis), where NF-κB-mediated inflammation is a key driver of thyroid follicular cell destruction(Zheng et al., 2022). Furthermore, chronic low-grade inflammation is a cornerstone of “insulin resistance in Type 2 Diabetes Mellitus (T2DM)”. The suppression of pro-inflammatory cytokines like IL-6 and TNF-α by Scrophulariae Radix components suggests a potential mechanism for improving insulin sensitivity(Sheng et al., 2024; Zhao et al., 2023).

### 3.2 Antioxidant effects

Flavonoids in *Scrophulariae Radix* help neutralize reactive oxygen species (ROS), protecting cells from oxidative stress (86, 87). Its polysaccharides increase the levels of antioxidant enzymes such as SOD and CAT, reducing free radical accumulation (88). In animal studies, these polysaccharides eased thyroid damage from radioactive iodine. They also sped up functional recovery, highlighting their potential in managing oxidative stress-related disorders.

### 3.3 Immunomodulatory effects

Polysaccharides from *Scrophulariae Radix* increase immune responses by stimulating macrophage and lymphocyte activity (89). They also activate NK cells, aiding in tumor suppression and immune balance (90), suggesting potential benefits for immune-related conditions such as thyroiditis and DM (29).

### 3.4 Cardiovascular protection

*Scrophulariae Radix* has shown notable cardioprotective effects in various studies (91). has reported that iridoid glycosides reduce cardiomyocyte apoptosis and improve ECG parameters in myocardial infarction models (92). has demonstrated that it reduces myocardial hypertrophy and fibrosis in ventricular remodeling (93). has revealed its antiremodeling effects by lowering the heart weight index and hormone levels in thyroxine- or aortic ligation-induced models (94). has reported that it alleviates remodeling in hypertensive rats by reducing blood pressure and cardiomyocyte apoptosis. These findings highlight its cardiovascular protective potential and the need for further research. The ability of Scrophulariae Radix to ameliorate myocardial remodeling and reduce apoptosis, as shown in these studies, suggests a direct application in managing hyperthyroidism-induced cardiac impairment. Similarly, cardiovascular disease is the leading cause of mortality in diabetic patients, highlighting the broad therapeutic value of this effect.

### 3.5 Antitumor effects

Polysaccharides from *Scrophularia ningpoensis* significantly increase the thymus and spleen indices and prolonge survival in S180 tumor-bearing mice (95). They also inhibite HaCaT cell proliferation and induce apoptosis, supporting their antitumor potential (96). Additionally, aucubin from *S. ningpoensis* suppressed A549 non-small cell lung cancer cell growth (97). The induction of apoptosis and inhibition of proliferation in various cancer cell lines, as demonstrated by its compounds like aucubin, provide a rationale for its investigation in thyroid carcinoma management.

### 3.6 Other pharmacological effects

*Scrophulariae Radix* also has antibacterial, hypoglycemic, and hepatoprotective effects. *In vitro*, acteoside improves primary hepatocyte viability and reduces LDH release. *In vivo*, it decreases the serum ALT and AST levels in acute liver injury models and exerts antiapoptotic effects by regulating the expression of apoptosis-related proteins, including Bcl-2 and Fas/FasL (Sheng et al., 2024; N. Zhang et al., 2023). The hypoglycemic effect is, of course, directly pertinent to DM treatment, forming the foundation for its use in blood glucose control. Simultaneously, the hepatoprotective effect is doubly relevant: it addresses hyperthyroidism-induced hepatic dysfunction and also protects against drug-induced or metabolic-associated liver injury in diabetic patients.

**Figure 1:**
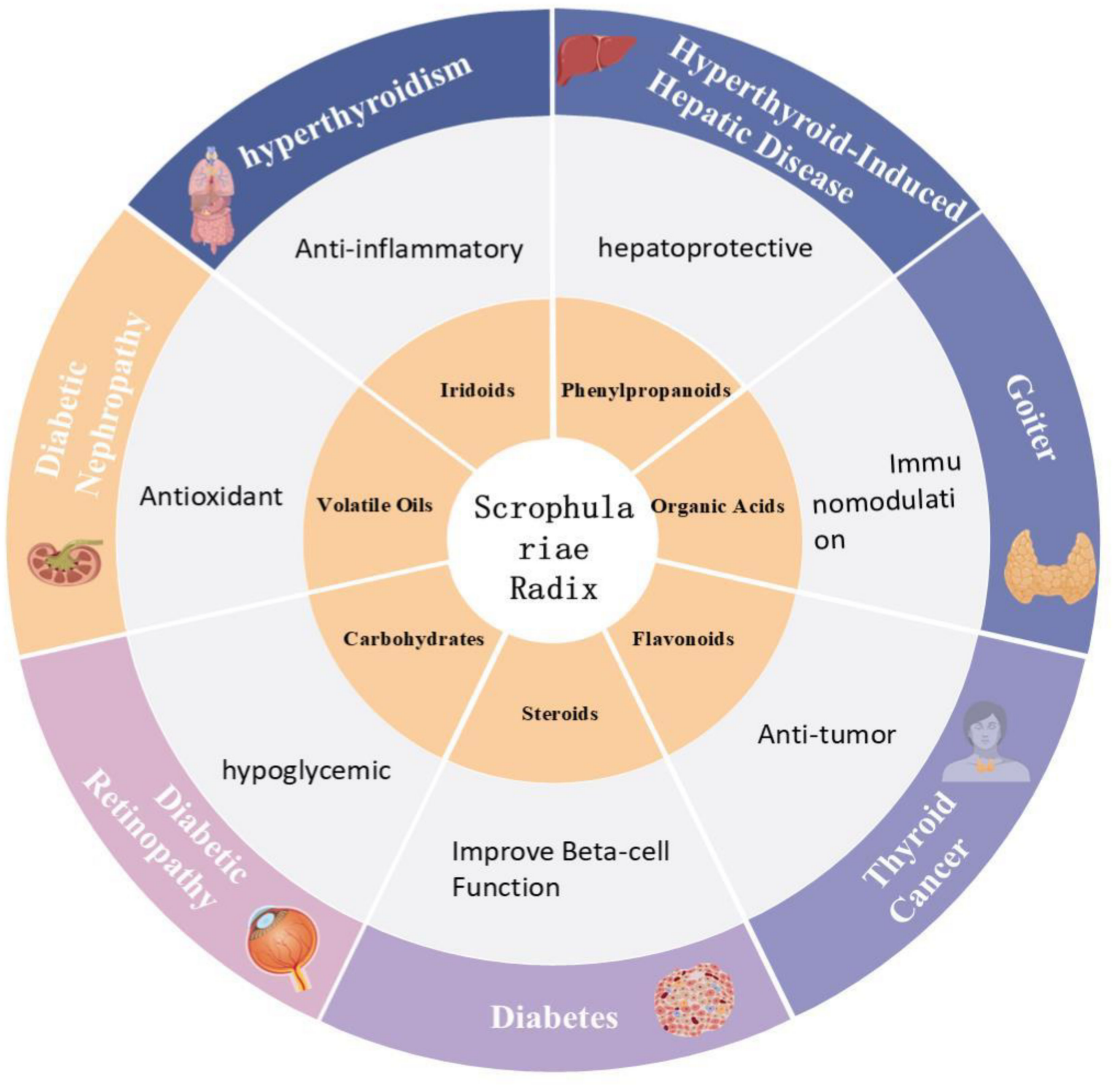
Information on the use of *Scrophulariae Radix* in the treatment of endocrine disorders.

In summary, the diverse compounds in *Scrophulariae Radix* exhibit multiple pharmacological activities relevant to thyroid diseases and DM through various mechanisms. These findings support its clinical potential and the development of new drugs, guiding further research on its role in these conditions. **Figure 1** summarizes its main compounds, pharmacological effects, and therapeutic applications in thyroid disorders and DM.

## 4 Application of *Scrophulariae Radix* in the treatment of thyroid diseases

### 4.1 Treatment of hyperthyroidism with *Scrophulariae Radix*

*Scrophulariae Radix* plays a crucial role in the treatment of hyperthyroidism by nourishing yin, reducing internal fire, softening hardness, and dispersing nodules, primarily acting on the liver meridian to regulate hyperthyroidism via hepatic pathways (98). Moreover, *Scrophulariae Radix* can soothe the liver, regulate qi, and calm liver yang. These actions promote the smooth flow of liver qi and help alleviate hyperthyroidism-related symptoms.

Studies have demonstrated that the ethanol extract of *Scrophulariae Radix* can markedly improve various abnormal indicators in a mouse model of hyperthyroidism induced by thyroid hormones, which simulates the syndrome of Yin deficiency and excessive internal heat. Improvements include significant recovery from weight loss, excessive food and water intake, and elevate body temperature (98, 99). report that *Scrophulariae Radix* modulates abnormal protein expression in rats with Yin deficiency and internal heat excess, mainly involving the ErbB signaling pathway.

In addition, *Scrophulariae Radix* inhibits abnormal thyroid cell proliferation by activating the MST1/Hippo pathway while promoting apoptosis and autophagy (100, 101). It downregulates cyclin D1 and upregulates caspase-3, preventing thyroid abnormalities and hormone imbalance. Enhanced autophagy facilitates the clearance of damaged cells, supporting thyroid structural stability. These mechanisms offer a novel pharmacological basis for hyperthyroidism treatment. Additionally, *Scrophulariae Radix* modulates key urinary, hepatic, and serum metabolites, helping to correct hyperthyroidism-related metabolic disturbances (102). They regulate the metabolic balance of tryptophan, tyrosine and other precursor molecules in urine, reduce the synthesis rate of thyroid hormones, and slow the imbalance in the body (101). With respect to liver metabolism, studies have shown that *Scrophulariae Radix* significantly regulates phospholipid metabolites such as phosphatidylcholine and phosphatidylethanolamine, aiding in restoring lipid metabolism balance and preventing common lipid metabolic disorders in hyperthyroid patients (102). In a rat model of hyperthyroidism induced by levothyroxine sodium, total extract and large polar iridoid fractions of *Scrophulariae Radix* significantly decrease key enzymes involved in glycolysis and the tricarboxylic acid cycle under hypermetabolic conditions (103). These compounds also inhibite glucokinase (GCK), pyruvate kinase (PK), and citrate synthase (CS) in the liver, further verifying the potential role of *Scrophulariae Radix* in regulating glucose metabolism and inhibiting excessive metabolism (103).

Scrophulariae Radix affects lipid metabolism. It modulates muscle lipases. Specifically, it increases adipose triglyceride lipase (ATGL) levels. As a result, it improves lipid metabolic disturbances linked to the hypermetabolic state of hyperthyroidism (104). These effects indicate that *Scrophulariae Radix* exerts systemic regulatory effects on thyroid hormone metabolism through multiple targets and pathways, optimizing energy and material metabolism and alleviating the metabolic burden in hyperthyroidism.

The active constituents of *Scrophulariae Radix*, such as phenylpropanoids and flavonoids, increase the activity of antioxidant enzymes (e.g., SOD and CAT) and protect thyroid cells from free radical-induced damage (105). Its extracts also suppress inflammatory mediators, including TNF-α and IL-1β, by downregulating inflammatory signaling pathways such as the ERK1/2 and JNK pathways. This anti-inflammatory action supports protection of the liver, cardiovascular system, and other vital organs in hyperthyroid conditions (100).

Hyperthyroidism is often accompanied by immune dysregulation. *Scrophulariae Radix* helps restore immune balance in the thyroid by reducing the levels of proinflammatory cytokines (e.g., IL-6 and TNF-α) and limiting excessive immune cell infiltration. Additionally, it suppresses the antiapoptotic protein Bcl-2 and upregulates the proapoptotic protein Bax, thereby inhibiting abnormal thyroid cell proliferation (101).

In summary, the therapeutic effects of *Scrophulariae Radix* on hyperthyroidism involve multiple pathways and multitarget regulation, suggesting new prospects for integrating TCM into modern thyroid disease management. These findings highlight its broad pharmacological potential and establish a scientific basis for future combined Chinese and Western therapeutic strategies.

### 4.2 Hyperthyroid-induced hepatic disease

Hyperthyroid-induced hepatic disease involves liver injury caused by excessive thyroid hormones, resulting in hepatocellular degeneration, necrosis, and hepatomegaly (106), and often presents with jaundice, elevated transaminases, or cirrhosis (107). Acteoside from *Scrophulariae Radix* has been shown to improve hepatocyte survival and reduce serum ALT and AST levels in rat liver failure models (108). Additionally, phenylethanoid glycosides inhibit hepatocyte apoptosis by upregulating *Bcl-2* and downregulating Fas/FasL signaling (109).

(100) has provided further mechanistic insights by employing UPLC-QE-HRMS, identifying 31 prototype compounds, 60 phase I metabolites, and 23 phase II metabolites of *Scrophulariae Radix in vivo*. Network pharmacology has predicted 96 potential molecular targets and relevant signaling pathways, with KEGG analysis highlighting Bcl-2, BAD, JNK, p38, and ERK1/2 as key nodes. Experimental validation has demonstrated that *Scrophulariae Radix* extract (XS), in combination with PTU, effectively reduced the elevated T3, T4, FT3, and FT4 levels induced by levothyroxine while increasing serum TSH levels. Additionally, it restore thyroid, liver, and kidney structure and function (100). These protective effects are attributed primarily to their antioxidative, anti-inflammatory, and antiapoptotic properties, providing scientific support for the use of TCM in thyroid disease management.

### 4.3 Treatment of thyroid cancer with *Scrophulariae Radix*

Thyroid cancer, a prevalent endocrine malignancy, arises from either follicular epithelial or parafollicular cells of the thyroid. It is classified into papillary, follicular, and anaplastic thyroid carcinomas, with papillary thyroid carcinoma accounting for approximately 85–90% of all cases (110). In its early stages, thyroid cancer is typically asymptomatic and is detected incidentally via painless cervical masses or nodules. As tumors progress, symptoms may develop. These include dyspnea, jugular vein distension, and hoarseness. Globally, thyroid cancer is the ninth most common cancer. In the United States, it is the seventh most common malignancy among women. It is also the most common cancer in adolescents and adults under 40 years of age (111). Notably, its incidence has risen sharply in recent years (110).

According to TCM, factors such as emotional stress, liver Qi stagnation, and phlegm-dampness accumulation contribute to thyroid cancer development (112). has assessed the cytotoxicity of *Scrophulariae Radix* on SW579 thyroid cancer cells via the MTT assay and determined the IC_50_ values to guide low-, medium-, and high-dose treatments for subsequent PCR analysis. The water extract of *Scrophularia ningpoensis* significantly inhibits SW579 cell growth and downregulates the oncogenes *Bcl-2* and *c-Myc*, key regulators of tumor progression.

### 4.4 Treatment of goiter with *Scrophulariae Radix*

Goiters, characterized by abnormal thyroid enlargement and symptoms such as dysphagia and dyspnea (113), arise from causes such as iodine deficiency, autoimmune disorders, thyroid nodules, and carcinoma (114). TCM is associated with Qi stagnation, blood stasis, and phlegm accumulation. *Scrophulariae Radix* is traditionally used for its lump-resolving and antigoiter properties.

Recent progress has been made in identifying the active constituents of TCM formulas for treating thyroid disorders. Serum samples from the model and treatment groups are analyzed using primary and secondary mass spectrometry. They are compared with the chemical profiles of the Scrophulariae Radix–Fritillaria combination. A total of 47 serum compounds are identified. Among them, 35 are derived from Scrophulariae Radix.

Propylthiouracil (PTU) is used to induce a rat goiter model. This allows further exploration of the therapeutic effect of the “Scrophularia Radix-Fritillaria” prescription. The experimental data show clear results. After treatment with “Scrophularia Radix-Fritillaria,” goiter symptoms in rats are significantly alleviated. The thyroid gland weight is markedly reduced. Serum thyroid hormone levels are effectively regulated and restored to the normal range (115). Histopathological analysis also shows notable improvements. After intervention with Scrophularia Radix-Fritillaria, the thyroid tissue structure improves significantly. This is reflected in the enlargement of the follicular cavity and the uniform distribution of colloids. These changes strongly suggest a protective effect on thyroid tissue. Further observations confirm these findings. The serum thyroid hormone (FT3, FT4) levels of rats in the Scrophularia Radix-Fritillaria group return to the normal range. TSH levels also decrease. These results are significantly different from those of the model group. Together, they verify the important role of Scrophularia Radix-Fritillaria in regulating thyroid function (115).

With advancements in modern medical technology, particularly in network pharmacology and multiomics technologies, research on the pharmacological mechanisms of *Scrophulariae Radix* is expected to expand further. These technological approaches aid in elucidating the multipathway, multitarget mechanisms involved in thyroid diseases, providing a theoretical basis for the development of new natural medicines. Future clinical and mechanistic studies can offer robust scientific evidence supporting the integration of TCM with Western medicine in thyroid disease treatment and potentially broaden the application of *Scrophulariae Radix* and its formulations to other endocrine disorders.

## 5 Application of *Scrophulariae Radix* in DM

### 5.1 Treatment of DM with *Scrophulariae Radix*

**Figure 2:**
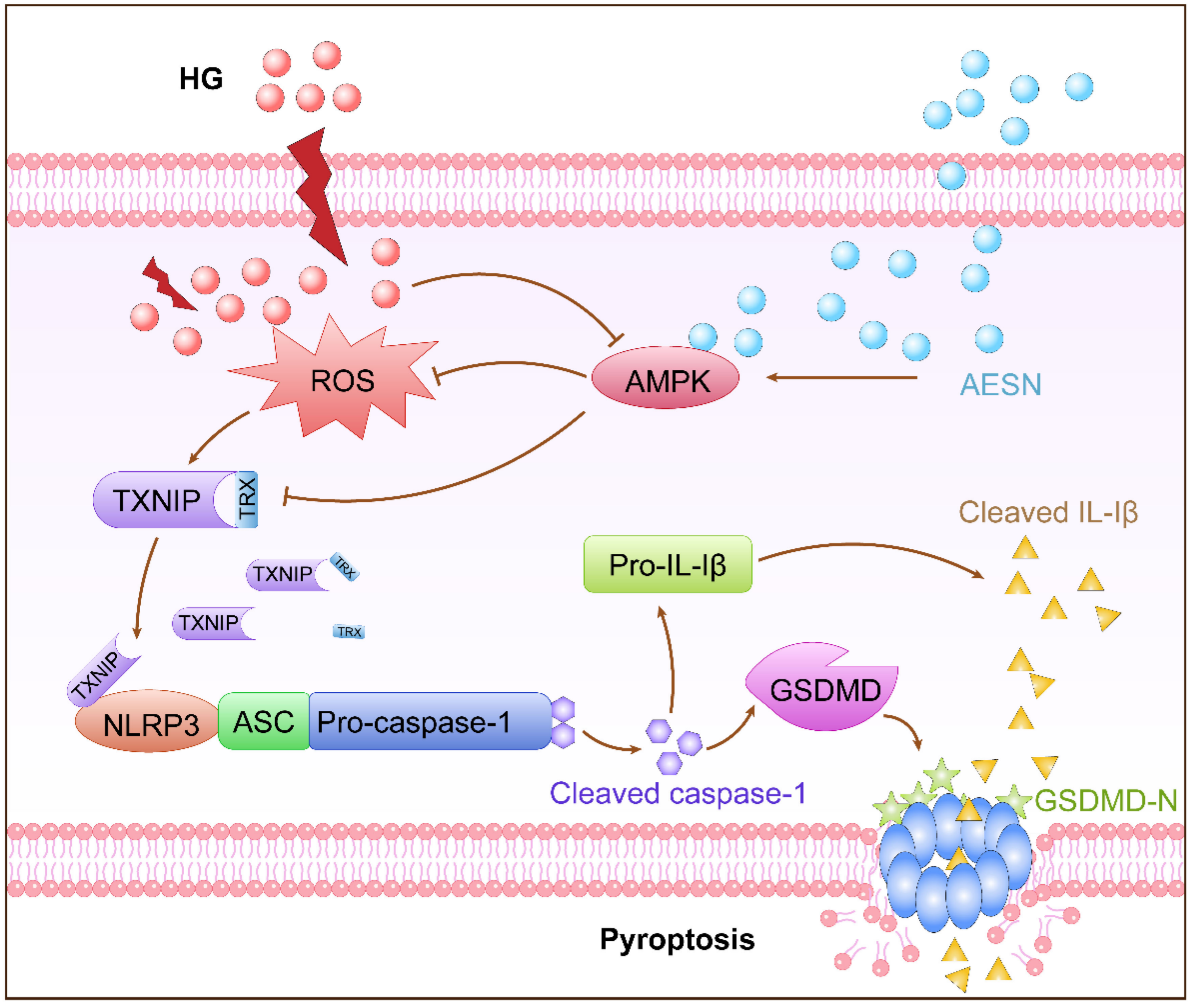
The mechanism of action of AESN in INS-1 cells.

The increasing global incidence of DM has posed substantial challenges to public health and significantly disrupted people’s daily lives (116). In TCM, *Scrophulariae Radix* is believed to treat diabetes mellitus (DM) by clearing heat, cooling the blood, nourishing yin, and reducing internal fire. Studies show that the aqueous extract of *Scrophulariae Radix* (AESN) alleviates insulitis in diabetic mice. It increases phosphorylated AMP-activated protein kinase (p-AMPK) expression in pancreatic tissues. At the same time, it suppresses NLRP3 inflammasome activity and gasdermin D (GSDMD) protein levels. These effects ultimately reduce β-cell apoptosis (117, 118). Additionally, AESN has been reported to activate INS-1 cells under high-glucose conditions (117), with the underlying mechanisms illustrated in **Figure 2**. DM leads to disordered glucose and lipid metabolism and decreased antioxidant capacity. However, *Scrophulariae Radix* polysaccharides can significantly increase the SOD level in model rats, which not only relieves diabetic symptoms but also enhances the body’s antioxidant capacity (119). Babaiedarzi et al. has used RT-PCR to detect the expression of *Pdx1* and *Ins1* genes. The results show that expression of both genes increase in diabetic rats treated with the *Scrophulariae Radix* ethanol extract (120). *Pdx1* is a key transcription factor for pancreatic development and β-cell function, while *Ins1* encodes insulin. The upregulation of these genes suggests improve pancreatic β-cell function, leading to enhanced insulin production and secretion (120). Network pharmacology research has identified the main active ingredients of *Scrophularia ningpoensis* in diabetes treatment. These include β-sitosterol, stigmasterol, taxifolin, harpagoside, and 14-deoxy-12(R)-andrographolide sulfonate. The study also identifies 14 common diabetes-related targets, such as prostaglandin endoperoxide synthase 2 (PTGS2), acetylcholinesterase (ACHE), and the β2-adrenergic receptor (ADRB2) (121). Molecular docking results further demonstrate distinct binding preferences. β-sitosterol binds strongly to glycogen synthase kinase-3β (GSK-3β). 14-deoxy-12(R)-andrographolide sulfonate binds strongly to PTGS2. Taxifolin also shows strong binding affinity for GSK-3β (121).

### 5.2 Treatment of diabetic retinopathy with *Scrophulariae Radix*

Diabetic retinopathy is the most common microvascular complication in diabetic patients and can lead to irreversible vision impairment, with severe cases resulting in complete vision loss (122). The pathogenesis of diabetic retinopathy is complex and involves multiple factors, such as blood vessels, nerves, and inflammation. It is widely regarded as a neurovascular disease (123).

Kan et al. has cultured human retinal microvascular endothelial cells (HRMECs) and has divided them into groups. These results show that *Scrophulariae Radix* extract may regulate HRMEC cell proliferation, migration, and apoptosis through the miR-646/VEGFA mechanism, improving diabetic retinal edema (124). The compound FXST, which contains *Scrophulariae Radix*, has been proven effective in treating diabetic retinopathy. FXST protects retinal endothelial cells from high glucose-induced damage through YAP-mediated effects and can also regulate the downregulation of endothelial growth factor expression (125, 126). Through these mechanisms, *Scrophulariae Radix* can protect the retina from damage caused by DM.

### 5.3 Treatment of diabetic nephropathy with *Scrophulariae Radix*

Diabetic nephropathy, which affects 20–40% of individuals with diabetes (127), is a major cause of end-stage renal disease and elevated cardiovascular risk (128). However, studies on *Scrophulariae Radix* in this context are limited (129). report that ethanol extracts suppressed renal expression of RAGE and S100A8, key mediators of inflammation and cellular injury in diabetic nephropathy, suggesting potential nephroprotective effects. Additionally, acteoside, a major active compound, has been reported to alleviate renal fibrosis and glycolipid metabolic disturbances by reducing oxidative stress and inflammation in β-cells, modulating the unfolded protein response, improving mitochondrial dynamics, and enhancing β-cell function and insulin secretion (69).

In conclusion, *Scrophulariae Radix* has multiple beneficial effects on the treatment of DM and its complications, including diabetic retinopathy and nephropathy. Nonetheless, further experimental studies and clinical trials are needed to elucidate its precise mechanisms of action and to identify its key bioactive constituents.

### 5.4 Treatment of diabetic foot with *Scrophularia radix*

Diabetic foot is one of the most common and life-threatening complications of diabetes, marked by high incidence, recurrence, and mortality (130–132). Luteolin, the core active component of *Scrophularia radix*, specifically activates AMP-activated protein kinase (AMPK), a key cellular energy sensor. AMPK phosphorylation regulates downstream targets, enhancing glucose uptake in skeletal muscle and other tissues. This process alleviates insulin resistance and systemic metabolic disorders, thereby creating a favorable metabolic foundation for diabetic foot ulcer repair (133). In addition, activation of this pathway suppresses NOD-like receptor protein 3 (NLRP3) inflammasome activity, reducing the maturation and release of proinflammatory cytokines such as interleukin-1β (IL-1β). This mechanism helps mitigate diabetes-associated chronic inflammation at the molecular level (134). From an immunoregulatory perspective, the *Scrophularia–Panax* compound (IST) modulates macrophage polarization by inhibiting differentiation toward the proinflammatory M1 phenotype. This modulation may influence the expression of key inflammatory mediators such as tumor necrosis factor-α (TNF-α), thereby improving the wound microenvironment in diabetes (134). Collectively, *Scrophularia radix* exerts synergistic effects on energy metabolism and inflammation suppression through the AMPK signaling pathway. These mechanisms provide a potential basis for alleviating the pathogenesis of diabetic foot.

### 5.5 *Scrophularia radix* in the treatment of diabetic cerebrovascular disease

*Scrophularia radix* demonstrates therapeutic potential in diabetic cerebrovascular disease, a major macrovascular complication of diabetes (135). Harpagoside, one of its active constituents, exerts neuroprotective effects by modulating endoplasmic reticulum–mitochondria coupling (MAMs), suppressing IP3R1/GRP75/VDAC1 complex expression, and maintaining neuronal calcium homeostasis. These actions prevent mitochondrial calcium overload and apoptosis (136). The aqueous extract of *Scrophularia radix* (RSAE) enhances antioxidant enzyme activities such as SOD and GSH-Px, reduces oxidative stress, regulates the Bcl-2/Bax balance, and inhibits MAPK phosphorylation. Collectively, these effects alleviate ischemia-reperfusion injury (137). In metabolic regulation, *Scrophularia*-containing formulations (e.g., Clean-DM1) activate IRS/PI3K/AKT and AMPK signaling pathways, thereby improving insulin resistance and correcting glucose-lipid metabolic dysfunction (138). The *Scrophularia–Atractylodes* herbal pair also modulates gut microbiota, enhances the efficacy of hypoglycemic agents, and indirectly protects brain tissue (139). With respect to vascular protection, *Scrophularia*-based prescriptions reduce stroke risk. A large cohort study reported a 33% lower incidence of stroke in diabetic patients receiving traditional Chinese medicine (adjusted HR = 0.67, 95% CI = 0.46–0.97). This protective effect is likely attributable to improvements in metabolic disturbances, inflammation, and oxidative stress (140). Overall, these findingsxindicate that *Scrophularia radix* exerts multi-component and multi-pathway regulatory effects, underscoring its integrative potential in the prevention and treatment of diabetic cerebrovascular complications.

In conclusion, *Scrophulariae Radix* has multiple beneficial effects on the treatment of DM and its complications, including chronic complications and the underlying metabolic dysregulation of diabetes. There is a notable lack of scientific literature exploring its potential effects on acute diabetic complications, such as diabetic ketoacidosis (DKA) or hyperosmolar hyperglycemic state (HHS). Investigating its role in these acute, life-threatening scenarios represents a critical and valuable direction for future research.

## 6 Clinical drug development potential of *Scrophularia radix* in thyroid diseases and diabetes

### 6.1 Potential of *Scrophularia radix* in clinical drug development for thyroid diseases

*Scrophularia radix* exhibits multifaceted potential in the treatment and drug development of thyroid disorders, particularly in compound formulations, active component discovery, and adjuvant therapy. In multi-herbal combinations, formulas containing *Scrophularia radix* and Panax ginseng (e.g., Isam-Tang) have demonstrated immunomodulatory and anti-apoptotic effects in experimental autoimmune thyroiditis models, alleviating thyroid inflammation and regulating proteins such as Bcl-2 and caspase-3, thereby indicating therapeutic prospects for autoimmune thyroiditis (141, 142). In hyperthyroidism models, Scrophularia-containing formulas modulate TSHR or hypothalamic–pituitary–thyroid axis function, effectively reducing T3 and T4 levels and promoting structural recovery of the thyroid gland (143–145). Active constituents such as harpagoside provide neuroprotection, suggesting indirect benefits in neuroendocrine disturbances associated with thyroid dysfunction (146, 147). In addition, the polyphenols enriched in *Scrophularia radix* may exert antioxidant effects and induce redifferentiation of thyroid cancer cells. For instance, curcumin has been shown to upregulate thyroid-specific transcription factors (TTF-1, TTF-2, PAX8) and restore NIS protein expression, thereby overcoming radioiodine (RAI) resistance and enhancing RAI therapy efficacy, offering new perspectives for thyroid cancer treatment (148, 149). Moreover, the traditional hepatoprotective properties of *Scrophularia radix* suggest a role as an adjuvant to mitigate hepatotoxicity during thyroid therapy and to potentiate the effects of antithyroid drugs such as methimazole (150, 151). Collectively, *Scrophularia radix* holds significant research value in compound development, mechanistic studies of its active constituents, and adjuvant applications, warranting further pharmacological and translational investigations.

### 6.2 Potential of *Scrophularia radix* in clinical drug development for diabetes

*Scrophularia radix* demonstrates unique advantages and promising prospects in the development of antidiabetic therapies. Its primary value lies in compound formulations. For example, the *Scrophularia–Panax* compound Isam-Tang (IST) exerts immunomodulatory effects, providing a foundation for novel therapies targeting immune regulation in diabetes (141). Compared with synthetic drugs, *Scrophularia radix*, as a natural extract, offers the advantage of low toxicity. This makes it a safe candidate for long-term use and aligns with holistic strategies for diabetes management (152, 153). Its therapeutic potential also derives from multitarget mechanisms. Rich in phenolic and flavonoid metabolites, *Scrophularia radix* inhibits α-glucosidase and α-amylase, activates the AMPK pathway, and regulates glucose metabolism while alleviating insulin resistance. These mechanistic features are well-suited to counteracting the complex pathology of diabetes (154–156). Furthermore, its antioxidant and anti-inflammatory properties provide additional benefits in preventing diabetic complications. Studies have shown reductions in C-reactive protein (CRP) and oxidative stress, suggesting protective effects in diabetic nephropathy and cardiovascular disease (135, 157). Nevertheless, the clinical translation of *Scrophularia radix* in diabetes remains limited, as most studies are preclinical and lack rigorous clinical validation. Overall, with its low toxicity, multitarget effects, and potential to mitigate complications, *Scrophularia radix* represents a promising natural candidate in antidiabetic drug development. Future research should prioritize identifying active constituents, elucidating precise mechanisms, and conducting standardized clinical trials to support its translational application.

## 7 Future prospects

Future research on *Scrophulariae Radix* should focus on several key areas:

### 7.1 Clinical trials and safety profiles

Comprehensive clinical trials are essential for assessing the efficacy, safety, and tolerability of *Scrophulariae Radix* in humans. Special attention should focus on its potential interactions with standard medications for thyroid disorders (e.g., methimazole, propranolol) and diabetes (e.g., metformin) to ensure safe integration into conventional therapies and support its rational clinical use.

### 7.2 Standardization and quality control

Given that the bioactive components of *Scrophulariae Radix* vary with plant origin, cultivation, and processing, standardized extraction methods and strict quality control are essential to ensure its stability, consistency, and therapeutic reliability for modern medical use.

## 8 Conclusion

In conclusion, *Scrophulariae Radix* shows strong potential as a natural treatment for endocrine disorders owing to its diverse bioactive compounds and multitarget effects. While TCM theory and preliminary studies offer a solid foundation, further well-designed clinical trials and molecular research are needed to confirm and expand its clinical applications.

As modern medical science continues to embrace integrative and holistic approaches, the role of *Scrophulariae Radix* in treating thyroid disease and managing DM exemplifies the potential synergy between traditional herbal medicine and contemporary medical research. This review underscores the importance of ongoing interdisciplinary research to develop new, effective, and safe therapeutic options for thyroid diseases, with *Scrophulariae Radix* positioned as a promising candidate for future clinical integration.
